# The creation of a healthy eating motivation score and its association with food choice and physical activity in a cross sectional sample of Irish adults

**DOI:** 10.1186/s12966-015-0234-0

**Published:** 2015-06-06

**Authors:** Paul Naughton, Sinéad N. McCarthy, Mary B. McCarthy

**Affiliations:** Food Market & Consumer Research Group, Department of Agrifood Business & Spatial Analysis, Teagasc Food Research Centre, Ashtown, Dublin, 15 Ireland; Department of Food Business and Development, University College Cork, Cork, Ireland; School of Agriculture, Food and Rural Development, Newcastle University, Newcastle Upon Tyne, NE1 7RU UK

**Keywords:** Food choice attitudes, Motivation, Activity, Dietary recommendation, Food consumption survey

## Abstract

**Background:**

This study aimed to develop a healthy eating motivation score and to determine if dietary, lifestyle and activity behaviours vary across levels of motivation to eat a healthy diet with a view to informing health promotion interventions.

**Methods:**

A cross-sectional survey of food intake, physical activity, lifestyles and food choice attitudes was conducted in a nationally representative sample of 1262 adults in the Republic of Ireland aged 18 years and over.

**Results:**

Increasing score for health motivation was significantly and positively related to healthy eating and exercise. Women, increasing age, normal BMI, regular exercise and increasing intakes of fruit and vegetables were associated with a higher odds ratio (OR) for having a high healthy eating motivation score. However, despite a high motivation score only 31 % of consumers in the strong motivation group achieved the recommendations for daily fruit and vegetable consumption, while 57 % achieved the fat recommendation. A higher intake of calorie dense foods from the top shelf of the food pyramid and increased time spent watching T.V. was associated with a decreased OR for positive motivation towards healthy eating.

**Conclusions:**

Healthy eating promotions directed at women and older adults should focus on supporting people’s motivations to attain a healthy diet by addressing issues such as dietary self-control and self-regulation. For men and younger adults, healthy eating promotions will need to address the issues underlying their weak attitudes towards healthy eating.

## Background

It has been long established by the world authorities such as World Health Organisation (WHO) that a poor quality diet and low levels of physical activity are causative factors in the development of many non-communicable diseases as well as having many social and economic consequences [1–3]. Some studies have cited that a positive change in health behaviour has the potential to reduce the global prevalence of many of these diseases by nearly 80 % [4, 5]. Indeed it has also been reported that disease burden can be dramatically reduced by decreasing six risk factors, all of which entail behaviour change [6]. Hence, policies that focus on improving dietary quality and increasing physical activity must incorporate programmes and interventions that take account of the complexities of human behaviour. This is especially important in relation to facilitating better food choice behaviours so that the healthier choice becomes an easier choice, an enjoyable choice and ultimately that it becomes the habitual choice for consumers. Policies and interventions to enhance diet and lifestyle have a diminished likelihood of success in the absence of an understanding of the motives underlying consumer behaviour and food choice in relation to a healthy diet and lifestyle.

For some time national dietary and lifestyle studies have clearly demonstrated that nutrient intakes and physical activity do not correspond with national guidelines in Ireland, United Kingdom and United States of America [7–9]. In developing strategies to promote healthy eating behaviour it is important to examine factors that influence food choice. An individual’s compliance with dietary recommendations is likely to be associated with their food choice attitudes. Attitudes are a fundamental component of behavioural motivation and are generally defined in terms of people’s overall evaluations (favourable or unfavourable) of performing a behaviour [10, 11]. Many of the influences on food choice, such as sensory preferences, beliefs about the nutritional quality and health effects of food, are likely to be accounted for by people’s dietary attitudes [12–14]. It is assumed that individuals engage in behaviours that support their attitudes [15]. For example, a person who has a strong positive healthy eating attitude would be expected to consume more fruit and vegetables than a person with a weak attitude. It is generally held that the direction (i.e. positive or negative) and the strength of peoples’ attitudes are determined by the interaction of instrumental beliefs (e.g. is performing the behaviour harmful-beneficial, valuable-worthless) and affective or experiential beliefs (e.g. is performing the behaviour pleasant-unpleasant, enjoyable-un-enjoyable) [14–16]. Previous research has shown that in general attitudes towards healthy eating are positive [17–21]. This can be attributed to the reasonably high level of nutritional knowledge held by adults in western societies [22–24]. However, the most recent national study into the food and nutrient intakes of Irish adults confirms that dietary recommendations do not coincide with actual food consumption patterns on a general population level [7]. This finding corresponds with research into the diets of adults in the UK and America [8, 9]. In developing strategies to promote healthy eating behaviour, it is important to examine factors that may influence food choice.

Therefore, from a health policy perspective, it is important to identify and also have an understanding of whether intentions to eat healthily, corresponds with actual eating behaviours. According to Eagly and Chaiken [13] high correlations between attitudes and behaviour should not be expected as there are many other important variables that guide overt behaviour. Nevertheless, research shows that attitudes indirectly influence behaviour through people’s behavioural motivations [25, 26]. The objective of this study is to develop a healthy eating motivation score and to determine if dietary, lifestyle and activity behaviours vary across different levels of motivation to eat a healthy diet with a view to informing health promotion interventions. Few studies have examined motivation-behaviour associations using comprehensive measures of food consumption [19]. Such studies are important in providing evidence based support for health policy initiatives directed at promoting healthy eating behaviours and increasing levels of physical activity.

Hence, in this study consumers are segmented based on their motivation to pursue a healthy diet. These segments are profiled in depth on diet, lifestyle and physical activity to provide a thorough understanding of consumer motivation and behaviour. In addition this research based evidence can support the development of consumer focussed health policy initiatives.

## Experimental methods

### Survey details

The National Adult Nutrition Survey (NANS) is a cross-sectional study of food and nutrient intakes, lifestyle choices, level of physical activity and food choice attitudes of Irish adults aged 18 years and over [7]. Extensive details of the survey and methodologies have been previously reported [7]. In summary, the survey was conducted in the Republic of Ireland during 2008–2011 on a representative sample of 1500 adults (740 males, 760 females). The response rate of the eligible sample was 60 %. Socio-demographic analysis and comparison of the sample to Census 2006 data as outlined in Table 1, has shown it to be representative of adults in Ireland with respect to age, gender, social class, and urban/rural location. From the sample of 1500, 1262 adults completed the food choice questionnaire, the main questionnaire used in this study. Ethical approval was granted by the University College Cork Clinical Research Ethics Committee of the Cork Teaching Hospitals and written informed consent was obtained from study participants.

**Table 1:** Socio-demographic breakdown of study sample and comparison with national Irish census data

		Census 2006	NANS dataset *n* = 1500	Study sample *n* = 1262
		%	%	%
Gender	Men	50	49	49
	Women	50	51	51
Age Group	18–35 years	35	35	38
	36–50 years	29	29	30
	51–64 years	21	20	20
	65+ years	15	15	12
Marital Status	Single	38	32	34
	Married/living with partner	51	58	58
	Widowed/separated/divorced	11	9	7
Location	Rural	39	30	28
	Urban	61	70	72
Social Class	Professional/managerial	33	45	46
	Non-manual	17	18	18
	Un/Semi/Skilled/manual	32	23	20
	Occupation Unknown/students	18	15	15

Food and beverage consumption was collected using a semi-weighed 4 day food diary. Respondents recorded detailed information regarding the amount and types of foods, beverages and nutritional supplements consumed over the recording period, as well as the cooking methods used, brand names of foods consumed and details of recipes. The food intake data was analysed using WISP (Weighted Intake Software Program; Tinuviel Software, Warrington, UK). WISP uses data from McCance and Widdowson’s The Composition of Foods and all nine supplemental volumes to generate nutrient intake data [27, 28]. Foods were grouped into the five shelves of the food pyramid, which is a graphic model for healthy eating guidelines as used by Irish national health promotion agencies and was recently revised by the Food Safety Authority of Ireland [29]. In summary, foods with a similar nutrient content are grouped on the same shelf. Foods from the top shelf are high in fat and/or sugar and should be used sparingly, ideally no more than one serving per day. The second shelf is high protein foods including meat, fish and eggs with recommended intakes of two portions per day. The third shelf is dairy foods with a recommendation of 3 servings per day. The fourth is fruit and vegetables, with a recommendation of more than five servings per day and the fifth is cereal based foods including breads, pasta and potatoes of which six servings per day is recommended.

Anthropometric measurements were measured by the researcher in the respondents’ homes. Height was measured to the nearest 0.1 cm using the Leicester portable height 6 measure (Chasmores Ltd, UK) with the respondents head positioned in the Frankfurt Plane. Weight was measured in duplicate using a Tanita body composition analyzer BC-420MA (Tanita Ltd, GB) to the nearest 0.1 kg after voiding, with light clothing and without shoes. Body Mass Index (BMI) was calculated as weight (kg) divided by height squared (m^2^) and was categorised according to the recommendations from the World Health Organisation [3]: normal (18.5–24.99 kg/m^2^), overweight (25.0–29.9 kg/m^2^) and obese (≥30 kg/m^2^). In this paper underweight respondents (<18.5 kg/m^2^) were not included in the analysis because they accounted for less than 1 % of the population.

In addition to the food diary, several questionnaires were completed by adults in the NANS, including Health & Lifestyle, Physical Activity and Food Choice Attitudinal questionnaires. In this paper, the analysis is primarily related to the food choice attitudinal questionnaire but also draws on data from the health & lifestyle and physical activity questionnaires. The health and lifestyle questionnaire provided information on respondents’ socio-demographics, education levels, supplement use, alcohol intake, and smoking status. The Epic Physical Activity Questionnaire (EPAQ2) was used to assess activity at home, work and recreation. The ‘Food Choice Attitudinal’ questionnaire was designed to measure people’s motivations and beliefs regarding food. In addition, the questionnaire provided measures of personal influences on food choice (e.g. habits, food neophobia, cooking skills) and societal influences on food choice (e.g. access to healthy foods). Wherever it was possible a range of measures from previously validated research instruments were used.

### Development of a construct to represent healthy food choice motivation

Food choice motives were measured in the questionnaire by taking a selection of items from the validated food choice questionnaire by Steptoe *et al.* and the Roininen *et al.* health and taste attitude scales (HTAS) [30, 17]. These five food choice motives included; health, taste, price, mood, and weight control (Table 2). The items were measured on a Likert scale from 1 strongly disagree to 7 strongly agree. Exploratory Factor analysis using principle components and orthogonal rotation (Varimax) was conducted in order to explore the dimensionality of healthy food choice motivation as well as the other food choice motives. The Kaiser-Meyer-Olkin measure was used to verify that the sample size was adequate for the analysis. KMO values between 0.5 and 1 indicate that factor analysis should yield distinct and reliable factors [31]. Bartlett’s test of sphericity was used to examine the inter-correlations between variables. Finally, items that had factor loadings of less than 0.4 and items that loaded across factors at ≥ 0.4 were removed and the analysis was rerun. Stevens [32] recommends using items of 0.4 or greater for interpreting factors. According to Costello and Osborne [33] the factor structure that best fits the data should have no or few item cross-loadings.

**Table 2:** Food choice questionnaire - motivation items

Motivation	Items
Health	It is important that the food I eat…
	Contains vitamins and minerals
	Keeps me healthy
	Is nutritious
	Is good for my appearance (skin/teeth/hair/nails etc.)
	I always follow a healthy and balanced diet
	I eat what I like and I do not worry about healthiness of food
	The healthiness of food has little impact on my food choices
Taste	It is important that the food I eat…
	Looks nice
	Tastes good
Price	It is important that the food I eat…
	Is not expensive
	Is cheap
	Is good value for money
Mood	It is important that the food I eat…
	Keeps me awake/alert
	Helps me cope with life
	Helps me relax
	Cheers me up and makes me feel good
	Helps me cope with stress
Weight Control	It is important that the food I eat…
	Helps me control my weight

### Statistical analysis

All statistical analysis and data manipulation were conducted using SPSS version 18 for Windows (SPSS, Inc. Chicago, IL). Cross tabulation analysis and the Pearson’s chi-squared test were used to examine significant differences in gender, age, social class, level of education and marital status across three healthy eating motivation groups. Differences in mean scores for the food consumption, dietary intakes, exercise, energy expenditure at work and the amount of time spent watching TV were analysed across the three motivation groupings using one-way analysis of variance. Gabriel’s pair-wise comparisons test was used to identify significant differences for the variables that complied with Levene’s test for homogeneity (*P* > 0.05). Where Levene’s test indicated that group variances were significantly different (*P* < 0.05) the Games-Howell procedure was used to identify where significant differences lay [31].

Multivariate logistic regression analysis was used to find the best fitting model describing the relationship between a dependent variable and a set of independent variables. The dependent variable was healthy eating motivation tertiles. Two of the three tertiles were used in the regression model to compare those with a strong healthy eating motivation to those with a weak healthy eating motivation score. Multivariate logistic regression was employed to determine which of the two categories a person belongs to given certain other information i.e. given the independent variables. The independent variables included socio-demographics, lifestyle factors and food consumption data. Within the multi variable logistic regression model the odds ratios reported for each independent variable are adjusted (i.e. they are adjusted odds ratios) as they account for the other variables (i.e. potential confounders) in the model. Pearson’s correlation analysis was carried out on the independent variables in order to assess the potential of multicollinearity in the data. According to Field [31] correlations below 0.8 are a good indication that multicollinearity is not occurring in the data. In addition, the variance inflation factor (VIF) indicates whether an independent variable has a strong linear relationship with other independent variables. According to Field [31] to be confident that multicollinearity is not biasing the regression model, all tolerance statistics should be at least 0.2 or above.

## Results

Seven of the items related to healthy eating motivation from Table 2 loaded together on one factor as presented in Table 3. The Kaiser-Meyer-Olkin was 0.836 and Bartlett’s test of sphericity was significant (*P ≤* 0.01) verifying that the sample was appropriate for factor analysis. Cronbach’s alpha for the seven healthy eating statements was 0.808 and the items-total correlations were all high. Item total correlations over 0.3 are considered good [31]. It is generally accepted that values over 0.8 represent a reliable scale [31, 34]. Furthermore, scale reliability in this study compares favourably with the study of Irish adults a decade previously by Hearty *et al.* [19] who obtained an alpha score of 0.71 for the three items measuring healthy eating motivation. The mean of the seven items representing healthy eating motivation was calculated to generate a healthy eating motivation construct for the total sample. In general, participants in the sample were positively orientated towards healthy eating with a mean score of 5.34 out of a maximum of 7. Respondents were then divided into three healthy eating groups (weak, moderate and strong) using the 33^rd^ and 66^th^ percentile points as cut off points for further characterisation (Table 4).

**Table 3:** Mean scores, factor loadings and item total correlation for the healthy eating motivation items

Items	$\overline{\mathrm{X}}$	SD	Factor loadings	Item-total correlation
It is important that the food I eat…				
Keeps me healthy	6.0	1.1	0.77	0.71
Is nutritious	6.1	1.0	0.73	0.61
Contains vitamins & minerals	5.6	1.3	0.73	0.54
Helps me control my weight	5.2	1.5	0.71	0.48
I always follow a healthy and balanced diet	4.4	1.5	0.64	0.50
**R** I eat what I like and I do not worry about healthiness of food	5.0	1.6	0.61	0.52
**R** The healthiness of food has little impact on my food choices	5.1	1.5	0.53	0.53

R indicates the statements that were reversed scored for analysis

**Table 4:** Mean scores for healthy eating motivation composite variable across the healthy eating motivation groups

Healthy eating motivation groups	*N*	$\overline{\mathrm{X}}$	SD	Minimum	Maximum
Weak motivation	475	4.4	0.7	2.0	5.1
Moderate motivation	424	5.6	0.2	5.2	5.9
Strong motivation	367	6.3	0.3	6.0	7.0
Total	1266	5.3	0.9	2.0	7.0

Analysis of the associations between healthy eating motivation and demographic characteristics was carried out using Pearson’s chi-square (Table 5). There was a significant association between gender and healthy eating motivation (*X*^2^ (2) = 34.852, *p = <* 0.001), with more males in the weak motivation group and more females in the strong motivation group. Furthermore, healthy eating motivation was significantly associated with age (*X*^2^ (6) = 49.558, *p = <* 0.001), marital status (*X*^2^ (4) = 32.288, *p = <* 0.001), social class (*X*^2^ (4) =15.946, *p = <* 0.001), and level of education (*X*^2^ (6) = 31.643, *p = <* 0.001). As Table 5 shows, there were a greater proportion of younger adults (18–35 year olds) and single adults in the weak motivation group. Adults over 51 years of age, married, in the professional, managerial and technical social classes with tertiary education were more likely to be in the strong healthy eating motivation group.

**Table 5:** Percentage of subjects with weak, moderate, or strong healthy eating motivations classified by demographic characteristics

	Population	Weak healthy eating motivation	Moderate healthy eating motivation	Strong healthy eating motivation
	%	%	%	%
Male	49.0	58.1	48.7	37.6
Female	51.0	41.9	51.3	62.4
Age: 18–35	38.3	48.4	36.3	27.5
36–50	30.2	28.0	31.8	31.1
51–64	19.6	15.8	20.4	23.7
65+	11.9	7.8	11.4	17.7
Marital status: Single	34.4	43.1	33.0	24.6
Married	57.9	49.9	58.9	67.2
Widowed/Separated/divorce	7.7	7.0	8.1	8.2
Social class: Prof/Mang/Technical	54.1	46.7	57.2	59.4
Non manual & Skilled	37.1	42.0	33.9	34.8
Semi-skilled/unskilled	8.8	11.2	8.9	5.8
Education: Primary	7.7	8.1	7.4	7.4
Intermediate	19.6	23.8	17	17.0
Secondary	24.2	28.7	24.7	17.9
Tertiary	48.6	39.5	50.8	57.7

Differences in food consumption and dietary intakes were examined across the healthy eating motivation groups using one-way analysis of variance (Table 6). Compared to the strong and moderate groups, fat made up a greater proportion of total energy for individuals in the weak healthy eating motivation group (*F* (2, 1260) = 8.537, *p <* 0.05). However, it is important to note that across the three groups, mean daily fat consumption as a proportion of total energy was above the 35 % limit recommended by European Food Safety Authority (EFSA). Even among people strongly motivated to eat a healthy diet, 43 % were not complying with EFSA’s dietary recommendation. Individuals in the strong healthy eating motivation group were consuming significantly more fruit and vegetables per day (*F* (2, 1263) = 68.432, *p <* 0.05) and significantly less foods high in fat and sugar compared to the weak group (*F* (2, 1263) = 10.378, *p <* 0.05). However, fruit and vegetable consumption across the three groups was less than the recommended target of ≥ 400 g per day proposed by the WHO. Even among people strongly motivated to eat a healthy diet, 69 % were not fully meeting this recommendation. The motivation groups were also profiled using lifestyle variables. Individuals in the weak and moderate groups expended more energy at work than individuals in the strong healthy eating motivation group (*F* (2, 1228) = 4.249 *p <* 0.05). However, individuals in strong healthy eating motivation group watched significantly less television during the week (*F* (2, 1250) = 18.336, *p <* 0.01).

**Table 6:** Mean scores of consumption and lifestyle factors across the healthy eating motivation groups

	Population		Weak healthy eating motivation		Moderate healthy eating motivation		Strong healthy eating motivation	
	$\overline{\mathrm{X}}$	SD	$\overline{\mathrm{X}}$	SD	$\overline{\mathrm{X}}$	SD	$\overline{\mathrm{X}}$	SD
% contribution of fat to total energy	34.6	6.1	35.5	6.3^d(a)^	34.1	5.9^d(b)^	33.9	6.1^d(b)^
Fats, sugary snacks (g/d)	75	53	83	59^d(a)^	71	51^d(b)^	68.5	44^d(b)^
Meat, fish, eggs (g/d)	227	104	232	109	224	94	225	108
Milk, cheese, yogurt (g/d)	275	201	260	197	290	215	279	190
Fruit and vegetables (g/d)	263	186	196	155^d^	273	177^d^	339	200^d^
Bread, cereals, potatoes (g/d)	355	149	346	149	364	156	357	141
BMI	26.9	4.9	27.1	5.1	27.1	5.2	26.4	4.4
Energy expenditure through work MET hrs/wk	90.9	60.3	64.8	61.6^d(a)^	58.7	61.5^d(a)^	52.4	56.5^d(b)^
Watching television hrs/week	19.6	10.0	21.7	10.8^d(a)^	18.8	9.3^d(b)^	17.7	9.3^d(b)^
Exercise minutes/wk	99.0	199.5	90.9	195.3	100.0	172.6	108.7	231.4
Complying with recommendations	%		%		%		%	
Fat ≤ 35 % of food energy	53		46		57		57	
Fruit and vegetables 400 g per day	21		11		23		31	

^abc^subscripts denote significant differences within groups

^d^indicates statistical difference

Multivariate logistic regression analysis was used to determine whether socio-demographic, consumption and lifestyle variables were significantly associated with healthy eating motivations as presented in Table 7. A binary dependent variable was used for this regression because the preceding analysis showed that the significant differences predominantly occurred between the strong and weak motivation groups. Pearson’s correlation analysis and the VIF were used to assess possible multicollinearity in the data. All correlations between the independent variables were less than 0.6 and all tolerance statistics were above 0.5 indicating that multicollinearity was not occurring in the data. As Table 7 depicts, women were two and half times more likely than men to have strong positive motivations towards eating a healthy diet (OR = 2.68, *p* < 0.01). Furthermore, the likelihood of being in the strong healthy eating motivation group increased with increasing age. Compared to the 18–35 year olds, older adults aged 51–64 were 2.7 times more likely to have strong healthy eating motivations (*p* < 0.01) and adults over 65 years old were 6 times more likely (*p* < 0.01). Obese respondents were less likely to have strong healthy eating motivations compared to respondents classified as normal weight (OR = 0.56, *p* = 0.05). Also, the likelihood of being strongly orientated towards healthy eating decreased with increasing levels of fat consumption as a proportion of total energy consumed (OR = 0.97, *p* = 0.05) and increasing intakes of snack foods high in sugar, fat and salt (OR = 0.65 *p* < 0.01). Whereas, the probability of being in the strong healthy eating motivation group increased with every median increase in daily intake of fruit and vegetables (OR =2.37, *p* < 0.01). Regarding lifestyle factors, there was a significant and positive relationship between exercise (i.e. time spent at vigorous recreational activities) and motivation to eat healthily. People who spent more than 120 mins per week exercising were nearly twice as likely to have strong healthy eating motivations (OR = 1.9, *p <* 0.05) compared to people who spent less than 26 mins per week exercising. Conversely, strong healthy eating motivation was negatively associated with time spent watching T.V. (OR = 0.82, *p* < 0.05).

**Table 7:** Multivariate logistic regression analysis of the influence of demographic, consumption and lifestyle factors on strong healthy eating motivation

Demographics	Odds ratio	95 % C.I. for odds ratio		Sig
		Lower	Upper	
Gender (Male)	2.68	1.64	4.34	0.00
Female				
Age (18–35)				0.00
36–50	1.70	0.98	2.95	0.06
51–64	2.70	1.41	5.16	0.00
65+	6.03	2.64	13.77	0.00
Social class (Prof/Mang/Tech)				0.37
Non manual & skilled	1.18	0.76	1.81	0.46
Semi-skilled/unskilled	0.70	0.33	1.49	0.35
Education (Primary)				0.11
Intermediate	1.70	0.74	3.88	0.21
Secondary	1.11	0.48	2.56	0.81
Tertiary	1.98	0.87	4.48	0.10
Marital status (Single)				0.20
Married	1.61	0.95	2.73	0.08
Widowed/separated/divorced	1.77	0.69	4.56	0.24
% contribution of fat to total energy	0.97	0.94	1.00	0.05
Fats, sugary snacks g/d^a^	0.65	0.50	0.83	0.00
Meat, fish, eggs g/d^a^	1.31	0.86	1.99	0.21
Milk, cheese, yogurt g/d^a^	1.05	0.82	1.33	0.72
Fruit and vegetables g/d^a^	2.07	1.58	2.72	0.00
Bread, cereals, potatoes g/d^a^	1.49	0.92	2.41	0.11
BMI (Normal)				0.08
Overweight	0.88	0.56	1.35	0.53
Obese	0.56	0.34	0.94	0.05
Exercise (< 26 min/wk)				0.05
Exercise 26–120 min/wk	1.41	0.87	2.25	0.16
Exercise > 120 min/wk	1.90	1.12	3.23	0.02
^b^Work Energy expenditure (low)				0.85
Medium work energy expenditure	0.88	0.55	1.43	0.62
High work energy expenditure	0.89	0.53	1.48	0.65
Total TV watched (hours/day)	0.82	0.71	0.94	0.01

Within the multi variable logistic regression model the odds ratios reported for each independent variable are adjusted as they account for the other variables in the model

^a^For better interpretation of the OR for the food intake data in this multiple regression model, new ORs and 95 % CIs values for a median increase of each food group (g/d) were calculated. The median value for each food group was 63 g/d fats and sugars; 214 g/d meat; 238 g/d milk & cheese; 230 g/d fruit & vegetables; 224 g/d breads & cereals

^b^Work energy expenditure in MET minutes per week: low ≤ 3374; medium 3375 – 5020; high ≥ 5021

## Discussion

In this study an examination of cross-sectional data into Irish adult’s dietary related motivations, food choices and lifestyle factors related to energy expenditure was undertaken. The findings indicate that healthy eating motivation is associated with healthy lifestyles. In general, people who were strongly motivated to eat a healthy diet had healthier dietary profiles, exercised more and watched less television compared to those with weak motivations towards eating a healthy diet. This study corresponds with previous research that has investigated the relationship between healthy motivations, consumption and lifestyle behaviours such as exercise [19, 35]. Furthermore, the findings provide support for the well established premise that healthy eating motivations are positively associated with healthy food choices [21, 36–38]. The analysis indicated that strong healthy eating motivation was associated with an increased intake of fruit and vegetables and a lower intake of foods high in fat. However, despite the better dietary profile and dietary behaviour, a proportion of the highly motivated group did not meet the dietary recommendations related to fruit and vegetable consumption and fat as a percentage of total energy. Thus, while individuals motivated to eat healthily are more likely to make healthy food choices, it appears that meeting dietary guidelines is nonetheless a challenge for some.

This study provides an analysis on the socio-demographic characteristics of Irish adults in relation to their motivation towards healthy eating. The logistic regression analysis indicated that there was a strong and significant association between increasing age and increasing health motivation. Furthermore, women were more likely than men to be positively motivated towards eating a healthy diet. These findings are consistent with previous studies [19, 39, 40]. Research shows that compared to men, women are more conscious of health when making food decisions and more likely to positively evaluate the benefits of eating a healthy diet [40–43]. A number of studies have found that healthy eating motivation becomes stronger with increasing age [17, 19, 44]. However, the finding that social class was non-significant was in contrast to previous studies where significant associations were shown between social class and healthy eating [19, 44]. Furthermore, an analysis of epidemiologic data (including the data used in this study) has shown that better quality diets are associated with higher social class status whereas the diets of people in the lower social classes are more often characterised as nutrient poor and energy dense [7, 45, 46]. It is possible that dietary discrepancies between social classes are not attributable to divergent motivations but are, perhaps, a consequence of some variables not measured in this study [47–49].

Over the last 10 years Irish adults have made little progress towards compliance with dietary guidelines [7, 49]. Yet it has been shown in this study and others that people’s motivations towards eating a healthy diet are generally positive [19]. There are a number of reasons that may account for this lack of convergence. The first is attitude ambivalence, which occurs when people have both positive and negative evaluations about behavioural performance [50–52]. Food is an area where individuals might be expected to be ambivalent [51, 53]. Research indicates that some people believe that healthy eating comes at the expense of other factors such as taste and convenience [54–56]. Therefore, healthy eating initiatives targeted at people with weak healthy eating motivations will need to consider issues underlying attitude ambivalence. For example, it may be necessary to address the taste and sensory characteristics of healthy food in order to introduce changes in motivation. A further issue that must be considered is optimistic bias, which refers to an individual’s belief that they are at less risk from various hazards compared to the average person [50]. In this regard, it may be necessary to continue to emphasise and clearly communicate the consequences and risks of unhealthy lifestyle choices in public health campaigns. The level of motivation to pursue a healthy diet may also be influenced by the stage of behaviour change. Some studies have shown that men were more likely to be in the pre-contemplative stage in terms of fat consumption compared to women who were more likely to be at the maintenance stage and hence had lower intakes of fat, whereas the men had not commenced any reduction strategies [40].

For people with strong motivations, especially those who were not meeting dietary guidelines, issues other than attitudes need to be considered. In many situations people’s behavioural decisions are not solely dependent on their motivations [57]. For example, people intend to eat healthfully, but perceive realistically that no healthy foods are available in the immediate food environment [58]. In this scenario, the individual does not perceive control over their behaviour. Therefore, healthy eating initiatives targeted at this cohort of people must facilitate dietary control by identifying and suggesting ways of overcoming barriers to healthy eating. This also implies that a healthy food environment needs to be established to keep these motivated individuals on a healthy trajectory. In addition, there is potential for social marketing to be employed in a multi-disciplinary approach to health promotion by providing key insights to overcome barriers and produce skills for health behaviour change [59].

It has been suggested that exercise may play a role in certain aspects of food choice [60]. Physical activity has been shown to be associated with better diet quality and higher fruit and vegetable consumption [61]. However, it remains unclear as to whether this healthier dietary behaviour is a consequence of being health orientated overall or whether it arises from biological and psychological consequences of activity. Nestle *et al.* [62] have also demonstrated that high levels of physical activity were associated with lower intakes of fat as a percentage of energy as well as long term weight loss maintenance. Hence it remains difficult to determine the cause or effect relationship between healthy eating motivation and physical activity.

A significant relationship between healthy eating motivation and food consumption has been observed in many studies [26, 63–66]. The majority of these studies have used food frequency questionnaires to measure food consumption behaviour and only a relatively small number of studies have examined dietary related motives and dietary intake using a food diary [19]. A food diary is a more precise means of dietary assessment and therefore, provides greater veracity to this observed motivation-behaviour relationship. When conducting such research it is important to consider the research design employed. The present study was cross-sectional (i.e. behaviour and motives were measured concurrently) and consequently it is not possible to draw casual conclusions on the relationship between motivation and behaviour [67]. However, reviewing past research and models of food choice suggests that motivation is an antecedent of behaviour [68–72]. In this regard, successful behavioural change would not be expected in the absence of changes in attitudes and motivations.

Accurate dietary intake is notoriously difficult to measure as indicated by doubly labelled water studies [73]. Within this study, food consumption data was measured using a semi-weighed four day food diary. Every effort was made to ensure that the respondent kept an accurate diary by a fieldworker visiting at the beginning, middle and end of the survey. This allowed for any potential gaps in the diary to be identified and rectified. However, by virtue of intervening in the respondent’s life, there is potential to alter eating habits in an attempt to portray a more healthy or desirable profile. To overcome this, each fieldworker emphasised the importance of maintaining current dietary patterns. Each of the respondents also completed the food choice questionnaire towards the end of the recording period. Hence, the subject was aware in terms of food and food choice motivations. This may be an advantage in that they are not passively ticking boxes on a questionnaire but rather had a more engaged process. The very nature of cross sectional data is a limit within its self in that cause or effect cannot be fully determined [63]. Rather it is just a snap shot of that moment in time of the person’s daily life. However, despite these limitations, the findings are supported by and comparable to many other studies in the literature.

## Conclusions

The findings from this study confirm that there is a positive and significant association between being strongly motivated to eat healthily and actually eating a healthy diet within a cross sectional sample of the Irish population. Furthermore, people who were strongly disposed towards eating a healthy diet spent more time exercising and less time watching television compared to people with weaker motivations. This motivation-behaviour association was most pronounced among women and became stronger with increasing age. In order to increase population compliance to dietary guidelines, it may be necessary to devise a dual strategy.

Policies and interventions to enhance diet and lifestyle will have an increased likelihood of success if the motives underlying behaviour and food choice are incorporated. Hence, strategies targeted at men and younger adults, should focus on motivational change and countering attitude ambivalence. Different strategies should take account of the differing motives in women and older adults. These strategies or interventions should support people who are motivated to eat healthily by addressing issues of dietary control and self-regulation.
